# Physicochemical and functional properties of *Lycium ruthenicum* pectin by different extraction methods

**DOI:** 10.3389/fnut.2022.946606

**Published:** 2022-08-09

**Authors:** Ziyang Wu, Dan Qin, Hehe Li, Dongqi Guo, Huan Cheng, Jinyuan Sun, Mingquan Huang, Xingqian Ye, Baoguo Sun

**Affiliations:** ^1^Zhejiang Key Laboratory for Agro-Food Processing, National-Local Joint Engineering Laboratory of Intelligent Food Technology and Equipment, College of Biosystems Engineering and Food Science, Zhejiang University, Hangzhou, China; ^2^Beijing Laboratory for Food Quality and Safety, Beijing Technology and Business University, Beijing, China; ^3^Key Laboratory of Brewing Molecular Engineering of China Light Industry, Beijing Technology and Business University, Beijing, China

**Keywords:** *Lycium ruthenicum*, pectin, extraction methods, physicochemical properties, functional properties

## Abstract

Three different extraction methods were used to extract high-temperature water-extracted pectin (HWp), high-temperature acid-extracted pectin (HAp), and high-temperature alkali-extracted pectin (HALp) from *Lycium ruthenicum*. The physicochemical properties, structure, and functional properties of three different pectins were studied. The results showed that HWp and HALp can extract rhamnogalacturonan-I (RG-I) from *L. ruthenicum* better. Through structural feature analysis, HWp and HALp have a branched structure, and HWp has a higher degree of esterification than HAp and HALp. Zeta potential results show that HWp solution is more stable. The thermal analysis results show that the thermal stability is HALp > HAp > HWp. HWp has the highest viscosity. The inhibitory activity results showed that HWp, HAp, and HALp have a certain inhibitory effect on α-glucosidase activity. This study shows the effects of different extraction methods on the properties of *L. ruthenicum* pectin and aims to provide a theoretical basis for the pharmaceutical and food industries to choose more suitable pectin extraction methods.

## Introduction

Pectin represents a group of structurally heterogeneous polysaccharides widely distributed in primary cell walls and the middle lamella of higher plants, which are rich in galacturonic acid (1). It is synthesized in the Golgi apparatus, released through exocytosis, and is commonly found in the primary wall of plant cell walls and the middle layer of cells. The pectin content in the primary cell wall of dicot plants is higher than that in the cell walls of gramineous plants. Pectin is considered to be a complex plant heteropolysaccharide. In some species, 35% of the primary cell wall is made up of pectin. Pectin is the most complex polysaccharide in nature and plays an important role in many industries (2). Commercial pectin consists of at least 65% galacturonic acid monomers. Commercial pectin is mainly composed of homogalacturonan (HG) with very low content of rhamnogalacturonan-I (RG-I). A more common method for producing pectin is to extract it using an acidic aqueous solution at 60–100°C under the condition of pH 1.5–3. The pectin thus extracted can achieve a more uniform quality. In the past few years, people have started to focus on the health properties of pectin. Studies have shown that chain conformation is an important parameter of polysaccharides and is closely related to the biological activity of polysaccharide (3). Therefore, the side chain of pectin has received extensive attention (4). Studies have shown that substituted rhamnogalacturonic acid (RGs) pectin can prevent cancer (5) by inducing apoptosis (6) in cancer cells. Through structural characterization, pectin has side chains and shows prebiotic potential.

*Lycium ruthenicum* Murr is a perennial wild thorny shrub belonging to the Solanaceae family, distributed in the salinized desert areas of northwest China (7). According to Chinese classical pharmacy books, the fruit of *L. ruthenicum* Murr is known by the common name “black wolfberry” in China. *L. ruthenicum* Murr is considered a traditional Chinese herbal remedy for menopause, menstrual irregularities, and heart disease (8). *L. ruthenicum* Murr has been studied in recent years because of its functional properties, and it has many functional components, such as pectin (9), flavonoids (10), anthocyanins (11), phenolic acid (12), alkaloids (13), and essential oils (14), which have good biological activity. The literature shows that pectin has antioxidant (15), hypoglycemic (16), and antifatigue activities (17). Nowadays, the structural and functional properties of pectin from *L. ruthenicum* have attracted extensive attention.

According to literature, different extraction methods result in different physicochemical properties and chemical structures of polysaccharides (18). Due to the extremely strong water solubility and alcohol insolubility of pectin, ethanol precipitation after extraction with water is the most commonly used extraction method in the extraction of pectin. This study aims to compare the structure and biological characteristics of pectin using three different extraction methods (water extraction, acid extraction, and alkaline extraction). Finding the difference between different extraction methods of pectin provides a certain theoretical basis for actual production.

## Materials and methods

### Materials and reagents

*Lycium ruthenicum* was purchased from Qinghai Province, China. All *L. ruthenicum* were dried and then grounded sufficiently into powder (50 mesh) and stored in desiccators until further use. Standard monosaccharides were purchased from Sigma-Aldrich (Shanghai) Trading Co., Ltd. (Shanghai, China). Ethanol (≥99%), HCl (37%), NaOH (≥99%) K_2_HPO_4_ (≥99%), and KH_2_PO_4_ (≥ 99%) were purchased from Sinopharm Chemical Reagent Co., Ltd. (Shanghai, China), 4-nitrobenzene-α-D-glucopyranoside (BR), α-glucosidase (≥99%), and acarbose (≥99%) were purchased from Mreda (Beijing, China). All ultrapure water and deionized water were obtained from Milli-Q system (Advantage A10, Billerica, United States).

### Extraction of *Lycium ruthenicum* pectin

*Lycium ruthenicum* consists of lots of phenolic compounds (19). The high-concentration alcohol solution can extract phenolic compounds well without destroying pectin or polysaccharides (20, 21). Therefore, *L. ruthenicum* powder was first treated with an 80% ethanol solution at 180 rpm for 48 h two times. The *L. ruthenicum* powder from which the free phenolic compounds were initially removed was obtained. Then, the obtained solid was degreased three times with n-hexane and dried at 40°C. The dried *L. ruthenicum* powder (30 g) was weighed and 900 ml deionized water was added. The high-temperature water extraction (HWE) directly extracted the sample after adding water. The high-temperature acid extraction (HAE) adjusted the sample with HCl (6 mol/L) to pH = 1. The high-temperature alkaline extraction (HALE) adjusted the sample with sodium hydroxide (NaOH) (6 mol/L) to pH = 13. Constant temperature heating magnetic stirrer (Yuhua Instrument Equipment Co., Ltd., Gongyi, China) was used to extract pectin at 85°C for 6 h, and then the extracted solution was cooled to room temperature. The pH of solutions that cooled to room temperature was adjusted to 7. Then, ethanol was added to the solution until the ethanol concentration was 80%. This solution was placed in a refrigerator at 4°C overnight. The overnight solution was centrifuged at 10,000 rpm for 15 min to obtain precipitated pectin. The supernatant was decanted, and the precipitate was dissolved with deionized water. The precipitate was placed in a water bath and heated at 70°C for 2 h to remove residual ethanol. Finally, the purified pectin was freeze-dried by freeze dryer (Alpha 1–4 LSCbasic, CHRIST, Osterode, Germany) for further analysis. The pectins obtained from HWE, HAE, and HALE were named HWp, HAp, and HALp, respectively.

The calculation method of the extraction rate is to divide the mass of the freeze-dried gum by the mass of the initial fruit powder.

### Physicochemical properties of *Lycium ruthenicum* pectin

#### Total sugar, total protein, and total phenols

For the sample chemical composition index, the total sugar, total protein, and total phenol of the sample were determined. The total sugar content was quantitatively determined using phenol-sulfuric acid colorimetric assay, in which glucose was used as a standard. The total protein content was determined using Bradford assay (Beyotime Biotechnology, China). The total phenolic content of the obtained samples was measured in gallic acid equivalent (GAE, μg/mg, dry weight basis) using the Folin–Ciocalteu colorimetric method.

### Monosaccharide composition

The monosaccharide contents of samples were calculated according to Hu et al. with small modifications (4). The sample (2 mg) was added to 4 M trifluoroacetic acid and hydrolyzed at 110°C for 8 h. Trifluoroacetic acid was removed by nitrogen blowing (25°C). After diluting the samples with ionized water, they were filtered and analyzed. Rhamnose (Rha), glucuronic acid (GluA), glucose (Glu), galacturonic acid (GalA), arabinose (Ara), galactose (Gal), fucose (Fuc), and xylose (Xyl) were used as a monosaccharide standard. The prepared samples and monosaccharide standard mixture were assayed at 30°C using a Dionex system (ICS-5000, Thermo Fisher, Waltham, MA, United States) with a pulsed electrochemical detector and a Carbopac™ PA10 analytical column. The separation was performed by isocratic elution with 18 mM NaOH for 15 min, followed by 18 mM NaOH in 100 mM NaOAc for the next 35 min.

### Molecular weight parameters

The molecular weight of the samples was determined using size-exclusion chromatography coupled to multi-angle laser light scattering. The molecules were separated by hydrodynamic volume (SEC column, OHpak SB-G guard column, SB-806 HQ and SB-804 HQ columns, 7.8 × 300 mm, Shodex, Japan). After the sample was separated in a column connected to an HPLC system, the molecules were passed through a MALS detector and detected by a laser beam. MALS quantifies and displays molecular weight by analyzing the signal and differential refractive index (dRI) signal. The specific parameters were elution with 0.15 M NaCl (pH 7.0) on the SEC column. The system run time was 90 min, and the sample injection volume was 100 μl. The dn/dc (refractive index increment) value was defined as 0.138 ml/g for all samples (22). All data were analyzed using the ASTRA software (7.1.2, Wyatt Technology, United States).

### Structural properties and stability of pectin

The structure of the samples was analyzed using Fourier transform infrared (FT-IR), and the pectin samples were ground with KBr and pressed into flakes for testing. Tablets were measured in the 4,000–400 cm^–1^ range using transmission mode and 32 scans on a spectrophotometer (Nicolet 5,700, Thermo Fisher Scientific, Waltham, United States). The calculation of the degree of esterification of pectin refers to the method of Monsoor (23). The peaks at 1,740 and 1,630 cm^–1^ in the infrared spectrum correspond to the C = O stretching vibrations of COOCH_3_ and COO^–^ on pectin GalA, respectively. The formula for calculating the degree of esterification (DE value) of pectin is as follows:


(1) $$DE\left(\%\right)=\frac{Area_{1740}}{Area_{1740}+Area_{1630}}\times 100$$

where Area_1740_ is the peak area of 1,740 cm^–1^ in the infrared absorbance spectrum, and Area_1630_ is the peak area of 1,630 cm^–1^ in the infrared absorbance spectrum. The analysis of the peak area was performed using OMNIC 7.3 (Thermo Fisher Scientific, Waltham, United States).

Congo red experiment was used to detect whether the pectin of different extraction methods had a triple-helix conformation. Congo red solution (80 μmol/L) and NaOH solution (1 mol/L) were configured. Then, 1 mg of HWp, HAp, and HALp and 2 ml of Congo red solution were added to different centrifuge tubes, respectively. The amounts of substances added to the NaOH solution were 0, 0.1, 0.2, 0.3, 0.4, and 0.5 mol/L, respectively. All solutions were diluted to 5 ml. Vortex shaker (IKA, Baden-Württemberg, Germany) was used to mix the solution and let it stand for 10 min at room temperature. After zeroing the UV-Vis spectrophotometer (UV-2700 220V CH, Shimadzu Instrument Mfg. Co., Ltd., Suzhou, China) with deionized water, the samples were scanned in the range of 400–600 nm. The maximum UV absorption wavelength of samples at different concentrations of NaOH was determined.

Zeta potential analysis was based on the classical dynamic light scattering theory. The zeta potential mode of the Malvern Zetasizer Pro laser particle size analyzer (Malvern, United Kingdom) was used to measure the zeta potential of the *L. ruthenicum* pectin solution. To avoid multiple scattering effects, 1% (w/v) pectin solution was diluted 100 times, and its potential was measured at 25°C.

### Atomic force microscopy and scanning electron microscope

The pectin samples were dissolved in deionized water to make a 1 mg/ml solution. The solutions were continuously stirred at 50°C for 12 h and then diluted to 10 μg/ml. The diluted pectin solutions (10 μl) were added dropwise on fresh matrix-cut mica and air-dried at 25°C. Atomic force microscope (AFM) images were collected with Cipher-S AFM (Asylum Research Instruments, United States) using a silicon cantilever (Si_3_N_4_) probe with a spring constant of 0.2 N/m and a resonance frequency of 13 kHz. Image analysis was performed using Nanoscope Analysis version 1.9 (Bruker, Rheinstetten, Germany).

Scanning electron microscope (SEM) (ZEISS, Oberkochen, Germany) was used to observe and take photograph of the surface microstructure of pectin under different extraction methods. All samples were fixed on the sample stage with double-sided sticky tape and covered with gold in a vacuum evaporator. Acceleration voltage was 3.0 kV, and the working distance was 10.6 mm. The surface and shape were collected at an appropriate magnification.

### Thermal analysis

For the determination of thermal stability of pectin samples, samples were characterized using a thermogravimetric analyzer (TGA 3+) (Mettler Toledo, Switzerland) in the temperature range of 50–600°C. Samples (3 mg) were analyzed in standard aluminum sample pans using high-purity N_2_ as the purge gas.

### Rheological characterization and dispersion stability

The rheological properties of the pectin samples were analyzed with an MCR302 Rheo-Stress rheometer (Anton Paar, Austria) with PP50 planar geometry. A 15% (w/v) solution of the sample was subjected to stable rate sweep testing over a shear rate range of 1–100 s^–1^ in logarithmic increments at 25°C. The data fit uses the following equation:


(2) $$\eta =\kappa \cdot \gamma^{\left(n-1\right)}$$

where η is the apparent viscosity (Pa⋅s), κ (Pa⋅sn) is the consistency index, γ is the shear rate (s-1), and n is the flow behavior index. Strain sweeps were used to determine the linear viscoelastic range. Changes in storage modulus (G′) and loss modulus (G″) of the mixed solution were measured over a frequency sweep from 1 to 100 Hz.

### Inhibition of α-glucosidase by *Lycium ruthenicum* pectin

α-Glucosidase inhibitors can competitively bind enzymes, inhibit the rate of carbohydrate hydrolysis in the intestinal tract, reduce the body’s absorption of carbohydrates, and achieve the purpose of controlling blood sugar. The inhibitory activity of HWp, HAp, and HALp on α-glucosidase was determined. The measurement method refers to the method of Lordan (24), and some adjustments have been made. The α-glucosidase solution (0.5 U/ml), 4-nitrophenyl α-D-glucopyranoside solution (5 mM), and HWp, HAp, and HALp pectin solutions (0.1, 0.2, 0.4, 0.6, 0.8, 1.0, and 2.0 mg/ml) were configured with a phosphate-buffered solution (0.1 M) with pH 6.9. The concentration of Na_2_CO_3_ solution was 0.2 M. Each reactant was added to a 96-well plate, and each group of experiments was repeated three times. Acarbose was used as a positive control.

The samples obtained in G_a_, G_b_, G_c_, and G_d_ measured the absorbance at 405 nm with a microplate reader (SpectraMax M2, Molecular Devices, San Jose, United States). The inhibitory efficiency of HWp, HAp, and HALp on glucosidase can be calculated according to the following formula:


(3) $$\mathrm{Inhibitory}\mathrm{efficiency}=\left[1-\frac{\left(G_{d}-G_{c}\right)}{\left(G_{b}-G_{a}\right)}\right]\times 100\%$$

## Results and discussion

### Physicochemical properties of high-temperature water-extracted pectin, high-temperature acid-extracted pectin, and high-temperature alkali-extracted pectin

#### Chemical composition

As shown in Table 1, the chemical compositions of HWp, HAp, and HALp were different. The yields of HAp (6.77 ± 0.54%) and HALp (6.19 ± 0.71%) are significantly higher than that of HWp (3.83 ± 0.73%), suggesting the contribution of acid or alkaline treatments in pectin extraction. This is due to the fact that acids and bases are good at breaking down the cell walls and releasing pectin. Compared with HWp (11.96 ± 1.65 μg GAE/mg), HAp (18.91 ± 1.69 μg GAE/mg) and HALp (17.09 ± 1.64 μg GAE/mg) had a higher phenolic content. Phenolic compounds are widely present in plants and are closely linked to plant cell walls through ester and ether bonds (20). The difference in total phenolic content among the three pectins may be due to the fact that the hydrogen bond between the cell wall and the polyphenols is broken under acidic or basic conditions, which leads to the release of more phenolic compounds (4). Compared with ordinary commercial pectin (1.46 μg GAE/mg pectin) and citrus pectin (5.89 ± 0.05 μg GAE/mg and 2.20 ± 0.16 μg GAE/mg) (25), the content of phenolic compounds in *L. ruthenicum* pectin is higher. This may be caused by the higher content of phenolic compounds in *L. ruthenicum* (26, 27). The total sugar content of HALp is significantly higher than that of HWp and HAp. The total protein content of HAp is significantly higher than that of HWp. Alkali-extracted pectin had more side chains, while acid-degraded pectin had more side chains. Given that arabinogalactose protein in *L. ruthenicum* pectin is an important glycoconjugate, a large part of the neutral sugar branch of RG-I in *L. ruthenicum* pectin should be tightly bound to the protein (28).

**TABLE 1:** Yields and the chemical compositions of HWp, HAp, and HALp.

	HWp	HAp	HALp
Yield (%, w/w)	3.83 ± 0.73^b^	6.77 ± 0.54^a^	6.19 ± 0.71^a^
Total sugar (%, w/w)	65.86 ± 1.74^b^	65.15 ± 2.68^b^	70.85 ± 1.17^a^
Total phenolic content (μg GAE/mg)	11.96 ± 1.65^b^	18.91 ± 1.69^a^	17.09 ± 1.64^a^
Total protein content (%, w/w)	4.77 ± 0.64^b^	5.82 ± 0.77^a^	6.43 ± 0.41^a^
**Monosaccharide (%)**			
Fuc	0.92 ± 0.15^a^	0.59 ± 0.11^a^	0.81 ± 0.01^a^
Rha	2.70 ± 1.45^b^	6.02 ± 0.54^ab^	8.12 ± 0.91^a^
Ara	38.16 ± 3.73^a^	0.81 ± 0.06^b^	38.31 ± 1.46^a^
Gal	20.67 ± 2.40^b^	26.94 ± 1.37^a^	18.46 ± 0.94^b^
Glu	5.35 ± 1.50^b^	11.40 ± 1.02^a^	3.00 ± 0.16^b^
Xyl	5.31 ± 1.90^b^	17.69 ± 4.29^a^	4.98 ± 0.50^b^
GalA	23.23 ± 4.77^b^	33.22 ± 0.37^a^	23.60 ± 0.77^b^
GluA	3.66 ± 0.32^a^	3.33 ± 0.13^a^	2.73 ± 0.03^a^
HG	20.53 ± 3.30^b^	27.20 ± 0.39^a^	15.48 ± 0.26^b^
RG-I	64.23 ± 9.03^a^	39.79 ± 0.23^b^	73.00 ± 4.21^a^
(Gal+Ara)/Rha	21.79 ± 2.27^a^	4.61 ± 0.24^c^	6.99 ± 0.30^b^

^*a,b*^Means that the data has significant differences.

The monosaccharide composition of the three pectin samples was determined by comparing the retention times of the chromatograms of the pectin samples with that of the monosaccharide standard. As shown in Table 1, all samples were composed of Fuc, Rha, Ara, Gal, Glu, Xyl, GalA, and GluA. HWE, HAE, and HALE will not change the type of monosaccharides, but the compositions of monosaccharides extracted from pectin by different extraction methods are different. The contents of GalA in the three pectin were relatively high, proving that they have better antioxidant activity (29). The content of GalA of HAp is significantly higher than those of HWp and HALp. The decrease in GalA content in pectin may be attributed to β-elimination reaction, which is one of the mechanisms of non-enzymatic degradation of pectin that will cleave the HG skeleton and de-esterify it when treated with alkaline medium (30). The pectin side chain is mainly composed of Ara and Gal (4). As shown in Table 1, the content of Gal of HAp is significantly higher than those of HWp and HALp. The Ara mol% of HWp and HALp was about 45 times higher than that in HAp. This phenomenon can be attributed to the degradation effect of acid treatment. Glu may come from residues of soluble sugars that were not completely removed during the extraction process (31). For acid hydrolysis, the content of Gal and Ara in acid degradation of citrus pectin decreases (32), which may be because Ara and Gal are the most unstable, while GalA is the most resistant to acid hydrolysis (33, 34). Therefore, the content of GalA in HAp is higher. For Fuc and GluA, different extraction methods have little effect on their contents. The molar ratio of (Gal + Ara)/Rha can demonstrate the contribution of the RG region in the pectin structure and the length of the branch connecting to RG-I (35). In this study, the molar ratios of (Gal + Ara)/Rha for HWp, HAp, and HALp were 21.79 ± 2.27, 4.61 ± 0.24, and 6.99 ± 0.30, respectively. The (Gal + Ara)/Rha molar ratios of HWp and HALp are high, hence the presence of long-chain branched RG-I domains in these two pectins. The molar percentage of HG in HAp was 27.20 ± 0.39 mol%, which was significantly higher than that in HWp and HALp. Therefore, the HG region of HAp dominates.

#### Molecular weight

The molecular weight of pectin plays an important role in the biological activity and use of pectin (36). Table 2 shows the molecular parameters of HWp, HAp, and HALp. For the three pectins, two symmetrical peaks were observed in the RI elution profile. In HWp and HAp, the concentration of the component with the highest molecular weight is lower, and the component with the lower molar mass is dominant. Compared with HWE, HAE can decompose pectin into smaller molar masses. In HALp, compared to HWp, HALE can decompose pectin into smaller molar masses, but 53.1% of pectin still maintains a high molar mass. These results showed that HWp, HAp, and HALp underwent degradation effect. Among them, HAp experienced the most serious degradation, followed by HALp. Although HWp has also undergone degradation, its effect is weaker than that of HAp and HALp. Polydispersity coefficients (Mw/Mn) of HALp (3.641 and 1.240) were a little higher than in HWp (1.464 and 1.084) and HAp (1.592 and 1.120). This indicates a broad molecular weight distribution of polysaccharides in HALp. The root mean square radii of the pectin samples from different treatments also varied, with values ranging from 25.600 to 138.900 nm. The above results indicate that the polysaccharides produced by the three treatment methods are all partially hydrolyzed. However, the polysaccharide sample treated with HALE maintains a higher molar mass.

**TABLE 2:** The molecular parameters of HWp, HAp, and HALp.

Mass fraction (%)	HWp		HAp		HALp	
	Peak 1	Peak 2	Peak 1	Peak 2	Peak 1	Peak 2
	34.8	65.2	19.7	80.3	53.1	46.9
Mw (g/mol)	(3.445 ± 0.029) × 10^6^	(1.271 ± 0.017) × 10^5^	(2.469 ± 0.028) × 10^5^	(3.973 ± 0.149) × 10^4^	(5.311 ± 0.020) × 10^6^	(9.440 ± 0185) × 10^4^
Mn (g/mol)	(2.353 ± 0.017) × 10^6^	(1.173 ± 0.017) × 10^5^	(1.551 ± 0.024) × 10^5^	(3.547 ± 0.147) × 10^4^	(1.459 ± 0.005) × 10^6^	(7.615 ± 0.143) × 10^4^
Polydispersity (Mw/Mn)	1.464 ± 0.016	1.084 ± 0.021	1.592 ± 0.030	1.120 ± 0.063	3.641 ± 0.019	1.240 ± 0.034
Rz (nm)	138.900 ± 0.972	38.400 ± 1.382	25.600 ± 1.536	44.600 ± 3.390	49.900 ± 0.349	52.700 ± 1.634

### Structural properties of pectin

#### Fourier transform infrared analysis

**FIGURE 1:**
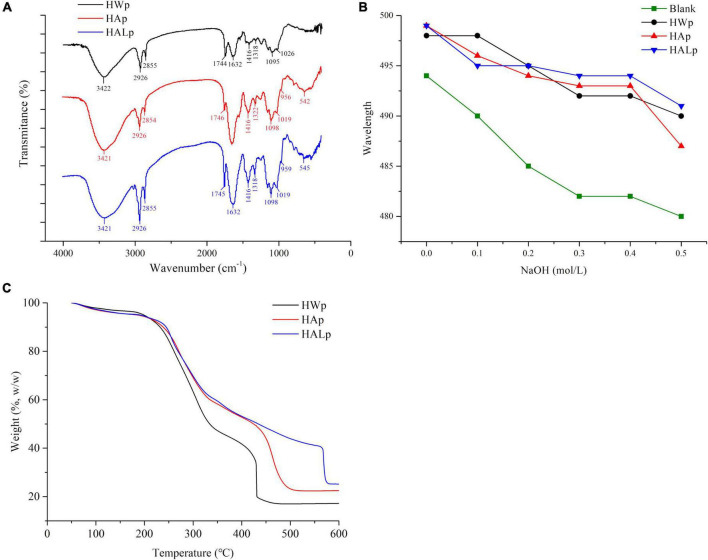
**(A)** FT-IR spectra of HWp, HAp, and HALp; **(B)** characterization of pectin helical structure at different concentrations; **(C)** thermodynamic properties of HWp, HAp, and HALp: TGA analysis.

As identified in Figure 1A, the three absorption bands appearing at 3,422, 3,421, and 3,421 cm^–1^ represent the stretching vibration of hydroxyl (-OH); the absorption band at 2,926 cm^–1^ represents stretching vibration of C–H. The absorption bands at 1,744, 1,745, and 1,746 cm^–1^ can be considered to be esterified carboxyl groups (COCH_3_), and the signals at 1,626 and 1,632 cm^–1^ show carboxylate groups. The degree of methoxylation (DM) of pectin can be determined from the percentage of peak area, which can be determined from the ratio of the area of the 1,740 cm^–1^ absorption band to the sum of the areas of the 1,740 and 1,633 cm^–1^ absorption bands (37). Figure 1A shows that HWp is a high methoxyl pectin, which is consistent with the results of the DM assay. The COO- absorption peaks at 1,746 and 1,626 cm^–1^ indicate that HALp has the lowest DM level. It may be that the de-esterification of alkali leads to the lower methoxy group of HALp, and the alkaline condition is the optimal condition for the production of low methoxyl pectin (30). The absorption peak of monosaccharides may exist below 1,100 cm^–1^. The characteristic peaks of β-galactan generally appear around 1,080 cm^–1^, while the characteristic peaks of arabinan mostly appear at 1,040 cm^–1^ (22). Furanose and pyranose rings may appear as peaks in the 920–950 cm^–1^ range. Above all, HALE can effectively reduce the level of DM.

According to Figure 1A and Formula (1), the degree of esterification of HWp is 31.03%, the degree of esterification of HAp is 2.96%, and the degree of esterification of HALp is 8.60%. Different extraction processes have a great influence on the degree of esterification, and the de-esterification effects of HAE and HALE are obvious.

#### Helical structure of pectin

Congo red is an acid dye that is soluble in water and ethanol. Congo red dyes can form complexes with polysaccharides with a three-strand helical chain conformation, and the ultraviolet maximum absorption wavelength of the complexes is red-shifted compared with Congo red solution. When the concentration of NaOH reaches a certain value, the maximum absorption wavelength will drop sharply. If the maximum absorption wavelength of the sample has a red shift, it proves that the sample has a helical structure. As shown in Figure 1B, compared with the Congo red solution, the UV maximum absorption wavelengths of HWp, HAp, and HALp all have a red shift, and they decrease as the concentration of NaOH increases. In summary, HWp, HAp, and HALp all have a triple helix structure.

#### Solution stability

Zeta potential refers to the potential difference between the surface of the dispersed droplets and the interior of the continuous phase in the emulsion, and is usually used to characterize the stability of the emulsion system. *L. ruthenicum* pectin is an anionic acidic polysaccharide that is negatively charged, and the measured potential of the emulsion is negative. The zeta potential of HWp is −46.07 ± 1.37, HAp is −35.08 ± 0.51, and HALp is −34.98 ± 0.95. The results show that the absolute value of the potential of HALp is the smallest, but there is no significant difference from HAp, and the absolute value of the zeta potential of HWp is large. This may be because HWE increases the degree of ionization of pectin, so that the surface of the solution droplets can absorb negatively charged molecules, the absolute value of the potential value increases, the mutual force is strong, and the solution is stable (38). Different extraction methods have significant effects on the zeta potential of *L. ruthenicum* pectin.

#### Thermal analysis

According to Figure 1C, the weight decrease trend of the three samples is similar. At the beginning, a slight weight loss was observed for the three pectins, and their weight remained largely unchanged before 250°C. At 200–250°C, the weight loss of the three pectins was mainly due to the loss of bound water in the pectin. Next, the weights of the three pectins dropped significantly during 250–350°C. The weight of HWp loss experienced slowed down in the range of 350–430°C and remain unchanged after 430°C. The weight of HAp loss experienced slowed down in the range of 350–500°C and remained unchanged after 500°C. The weight of HALp loss experienced slowed down in the range of 350–570°C and remained unchanged after 570°C. During the rapid weight loss phase, the weight loss onset for HWp, HAp, and HALp at 215.1, 232.6, and 233.4°C was caused by polysaccharide pyrolytic decompositions. At this time, the three kinds of pectins were thermally degraded under the action of temperature, the polysaccharide chain was decomposed, the acid group was decarboxylated, and the carbon–carbon double bond in the pyran ring was decomposed (39). Extensive pyrolysis of carbon occurs at 400–600°C. According to Figure 1C, the total mass loss of HWp is 82.8%, that of HAp is 77.5%, and that of HALp is 74.8%. Compared with HAE and HWE, the pectin samples prepared by HALE have better thermal stability, which can provide certain data support for choosing different pectin extraction method in the future.

#### Atomic force microscope and scanning electron microscope imaging of high-temperature water-extracted pectin, high-temperature acid-extracted pectin, and high-temperature alkali-extracted pectin

**FIGURE 2:**
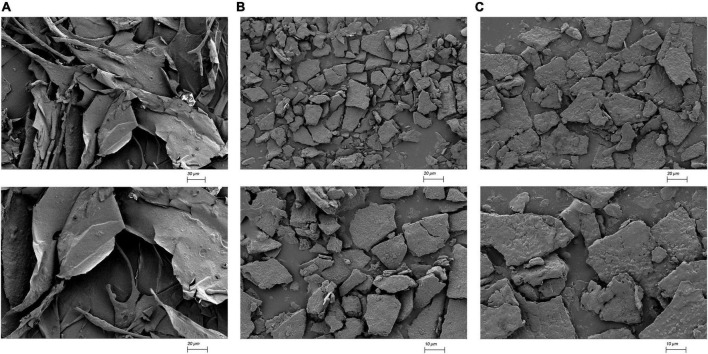
SEM imaging of HWp **(A)**, HAp **(B)**, and HALp **(C)**.

As shown in Figure 2, the results showed that the different material induced different physical changes. The microstructure of HWp is smooth and thin. The microstructure of HAp is relatively smooth and finely fragmented. The microstructure of HALp is a large block with irregular particles on the surface. The dense structure of HWEs causes them to cross-link with each other. This is also the reason for the high apparent viscosity of HWE. For HAE and HALE, the sheet-like structure is in a dispersed and stretched state, resulting in a low overall viscosity of the solution.

**FIGURE 3:**
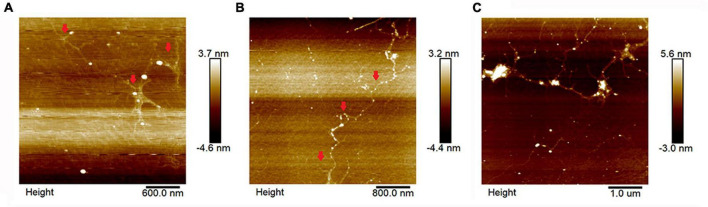
AFM imaging of HWp **(A)**, HAp **(B)**, and HALp **(C)**.

AFM can be used to visually observe the surface morphology of pectin samples. As shown in Figure 3, HWp and HALp are mainly a branched structure, while HAp is a chain structure. HWp, HAp, and HALp displayed heights of 2.3, 2.0, and 2.8 nm, respectively, on the mica flakes, which indicated that the polysaccharide chains of pectin were also slightly aggregated at low concentrations and would not be completely decomposed. Compared with the three pectins in Figure 3, HWp and HALp have branched structure, and HAp mainly has a linear structure, suggesting that HWE and HALE may lead to a high RG-I pectin region content.

### Rheological characterization

**FIGURE 4:**
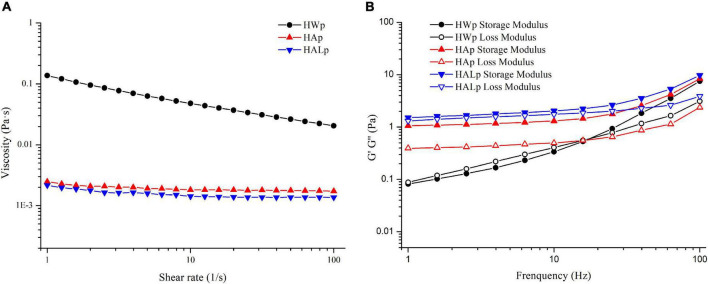
Rheological characterization of HWp, HAp, and HALp: **(A)** flow behavior and **(B)** frequency sweep of modulus G′ and G″.

The change in apparent viscosity with shear rate is illustrated in Figure 4A. The apparent viscosity of the three pectin solutions decreased with increasing shear stress (1–100 s^–1^), so the samples all behaved as non-Newtonian pseudoplastic fluids. Other sources of pectin, such as those from apple peels (40) and lime peel (41), exhibited similar properties. Table 3 shows the specific parameters of the power-law model constructed according to Equation (2), with larger κ values reflecting the increase in flow resistance and viscosity. By eye observation, the viscosity of the three pectin solutions was the largest in HWp and the smallest in HALp. The molar mass parameters, substituent distribution, and branching together determine the rheological properties of pectin solutions (42). In this study, the branched structure of HWp may have contributed to the high viscosity of the solution.

**TABLE 3:** Parameters of flow curves obtained by fitting to power law model.

Sample	Concentration (w/v %)	κ (Pa⋅s*^n^*)	*n*	*R* ^2^
HWp	15%	0.126	0.595	0.996
HAp	15%	0.002	0.913	0.830
HALp	15%	0.002	0.938	0.823

Storage modulus G′ refers to the ability of a viscoelastic material to store energy in a cycle under the action of alternating stress, usually referring to elasticity; energy dissipation modulus G″ refers to the ability to consume energy in a changing cycle, usually referring to viscosity. The dynamic viscoelastic properties of HWp, HAp, and HALp are shown in Figure 4B. The degree of elastic deformation of HAp is higher than that of HWp and HALp. Both HAp and HALp at 15 w/v% appeared as thick liquids and could be defined as weak gels as G′ > G″ (43). This result shows that the molecular chain entanglement of pectin at this time belongs to the gel system, the elasticity is strong, and the pectin is mainly elastic. The intersection of the storage modulus and loss modulus of HWp indicates that HWE can enhance the gelation of pectin. It may be due to the exposure of many connecting regions during the process of HWE extraction of pectin, the interaction of pectin chains is greater, and the cross-linking system formed is stronger, showing typically dynamic rheological properties of gel. It may be that the high RG-I domains lead to the creation of branched structures, resulting in the rheological properties of HWp. This has also been reported in other pectins rich in RG-I (44). The branched structure of the pectin extracted with HALE may be destroyed by the alkali solution, resulting in a less viscous solution, which is consistent with the molecular weight data.

### Inhibit enzyme activity

**FIGURE 5:**
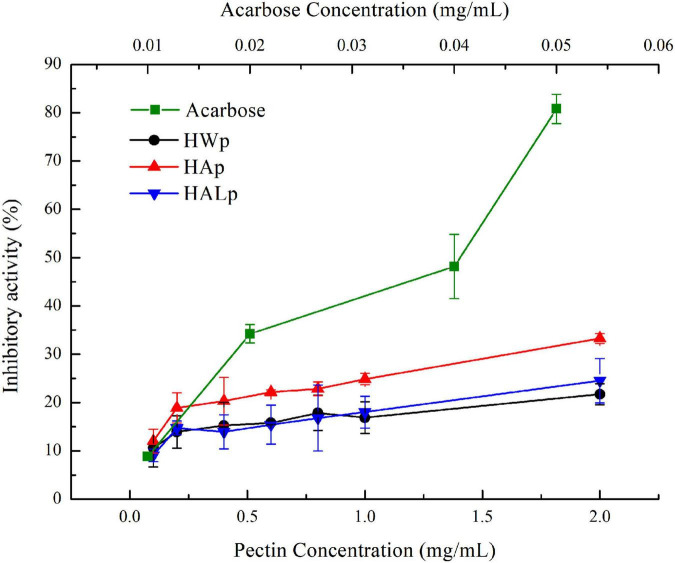
Different concentrations of HWp, HAp, HALp, and acarbose glucosidase inhibition efficiency.

As shown in Figure 5, different concentrations of HWp, HAp, and HALp reacted with α-glucosidase. Among them, the inhibition of α-glucosidase by HWp, HAp, and HALp was in a significant dose-dependent relationship, and the inhibition efficiency increased as the concentration increased. When the concentration of HWp, HAp, and HALp was 2 mg/ml, the inhibitory efficiency of α-glucosidase reached 21.73 ± 2.19%, 33.28 ± 1.03%, and 24.54 ± 4.60%, respectively. Compared with acarbose, HWp, HAp, and HALp have inhibitory effects on α-glucosidase. When the concentration of acarbose is 0.05 mg/ml, the inhibitory efficiency of α-glucosidase is 80.80%. Therefore, HWp, HAp, and HALp have a certain inhibitory effect on α-glucosidase activity, but the inhibitory intensity is much smaller than the control acarbose, and the order of the inhibitory intensity is HWp < HALp < HAp < acarbose.

## Conclusion

Three different extraction methods were used to extract HWp, HAp, and HALp from *L. ruthenicum*. The yields of HAp (6.77%) and HALp (6.19%) are significantly higher than HWp (3.83%). Analysis of monosaccharide composition shows that the Ara content of HALp and HWp is significantly higher than that of HAp, and the Gal, Glu, xyl, and GalA contents of HAp are significantly higher than that of HWp and HALp. The RG-I contents of HALp (73.00%) and HWp (64.23%) are significantly higher than that of HAp (39.79%). The results show that HWE and HALE can extract RG-I from *L. ruthenicum* better. The physicochemical properties, structure, and functional properties of three different pectins were studied. FT-IR results show that different extraction processes have a great influence on the degree of esterification, and the de-esterification effects of HAE and HALE are obvious. AFM results show that HWp and HALp have a branched structure, and HAp has a chain structure. The crystallinities of the three pectins are similar. Zeta potential results show that HWp solution is more stable. The thermal analysis results show that the thermal stability is HALp > HAp > HWp. The rheological properties of the extracted pectin were studied. The dynamic modulus of HWp is more dependent on frequency, indicating that the pectin obtained by HAE is more liquid-like than HAp and HALp. HWp has the highest viscosity. The inhibitory activity results showed that HWp, HAp, and HALp have a certain inhibitory effect on α-glucosidase activity. This study provides new insights into the impact of different extraction methods on pectin and aims to provide a theoretical basis for the pharmaceutical and food industries to choose more suitable pectin extraction methods.

## Data availability statement

The raw data supporting the conclusions of this article will be made available by the authors, without undue reservation.

## Author contributions

ZW: methodology, software, and writing—original draft. DQ: methodology and software. HL: conceptualization and funding acquisition. DG: methodology. HC: funding acquisition. JS and MH: writing—review and editing. XY and BS: supervision and funding acquisition. All authors contributed to the article and approved the submitted version.
